# The impact of flooding on people living with HIV: a case study from the Ohangwena Region, Namibia

**DOI:** 10.3402/gha.v8.26441

**Published:** 2015-03-25

**Authors:** Carmen Anthonj, Odon T. Nkongolo, Peter Schmitz, Johannes N. Hango, Thomas Kistemann

**Affiliations:** 1 Institute for Hygiene and Public Health, WHO CC for Health Promoting Water Management and Risk Communication, University of Bonn, Bonn, Germany; 2 Directorate of Special Programmes, Ministry of Health and Social Services (Ohangwena Region), Eenhana, Namibia

**Keywords:** disaster preparedness, flooding, food security, HIV and AIDS, Namibia, vulnerability

## Abstract

**Background:**

Floods are a disaster situation for all affected populations and especially for vulnerable groups within communities such as children, orphans, women, and people with chronic diseases such as HIV and AIDS. They need functioning health care, sanitation and hygiene, safe water, and healthy food supply, and are critically dependent on their social care and support networks. A study carried out in the Ohangwena region, Namibia, where HIV prevalence is high and extensive flooding frequently occurs, aims to provide a deeper understanding of the impact that flooding has on people living with HIV (PLWHIV) as well as on HIV service providers in the region.

**Design:**

The qualitative research applying grounded theory included semi-structured interviews with PLWHIV, focus group discussions with HIV service providers, and a national feedback meeting. The findings were interpreted using the sustainable livelihoods framework, the natural hazard research approach, and health behaviour theories.

**Results:**

The study reveals that flooding poses major problems to PLWHIV in terms of their everyday lives, affecting livelihoods, work, income, and living conditions. The factors threatening them under normal conditions – poverty, malnutrition, unsafe water, sanitation and hygiene, limited access to health facilities, a weak health status, and stigma – are intensified by flood-related breakdown of infrastructure, insecurity, malnutrition, and diseases evolving over the course of a flood. A potential dual risk exists for their health: the increased risk both of infection and disease due to the inaccessibility of health services and antiretroviral treatment. A *HIV and Flooding Framework* was developed to display the results.

**Conclusions:**

This study demonstrates that vulnerabilities and health risks of PLWHIV will increase in a disaster situation like flooding if access to HIV prevention, treatment, care and support are not addressed and ensured. The findings and the *HIV and Flooding Framework* are not specific to Ohangwena and can be transferred to any flood-affected region that has a high HIV prevalence and relies mainly on subsistence agriculture. They serve as a model case for analysing vulnerabilities related to health and health service provision under disaster conditions. The impact will vary according to the physical, geographical, climatological, social, and behavioural characteristics of the region and the people affected. In the Ohangwena region, a disaster risk management mechanism is already in place which addresses people with HIV during flooding. However, preparedness could be improved further by applying the *HIV and Flooding Framework*.

HIV and AIDS are part of people's everyday lives in Namibia. Official estimates indicate that Namibia is one of the countries most affected by the epidemic with one of the highest HIV prevalence worldwide. In Namibia, 13.5% of the adult population and 2.1% of the children, or about 188,000 people, are HIV-positive. Annually, there are 4,500 AIDS-related deaths and the consequences manifest themselves in all segments of society and the economy. About 99,600 people infected were in need of antiretroviral treatment (ART) in 2011, with 88,717 people receiving medication (1). In Namibia, there were about 12,000 notified cases of TB in 2011, with 50% of them being HIV-positive and of these, 54% on ART (2). The high HIV mortality rate and the large number of orphans have a negative effect on demographic development. Due to the epidemic, people cannot maintain their jobs, thus the economy as well as public services declines. Important institutions, including the health sector, are constantly under pressure and are unable to respond adequately. Societies and social networks fall apart and individuals have difficulty in sustaining their livelihoods and living healthily. In particular, nutrition and access to health services are challenging. Women and children, especially orphans, belong to the high-risk group for HIV and are the hardest hit (3). The northern regions of Namibia, including Ohangwena, report the highest HIV prevalence rates in the country, especially among women aged 25–49 (Fig. 1).

**Fig. 1:**
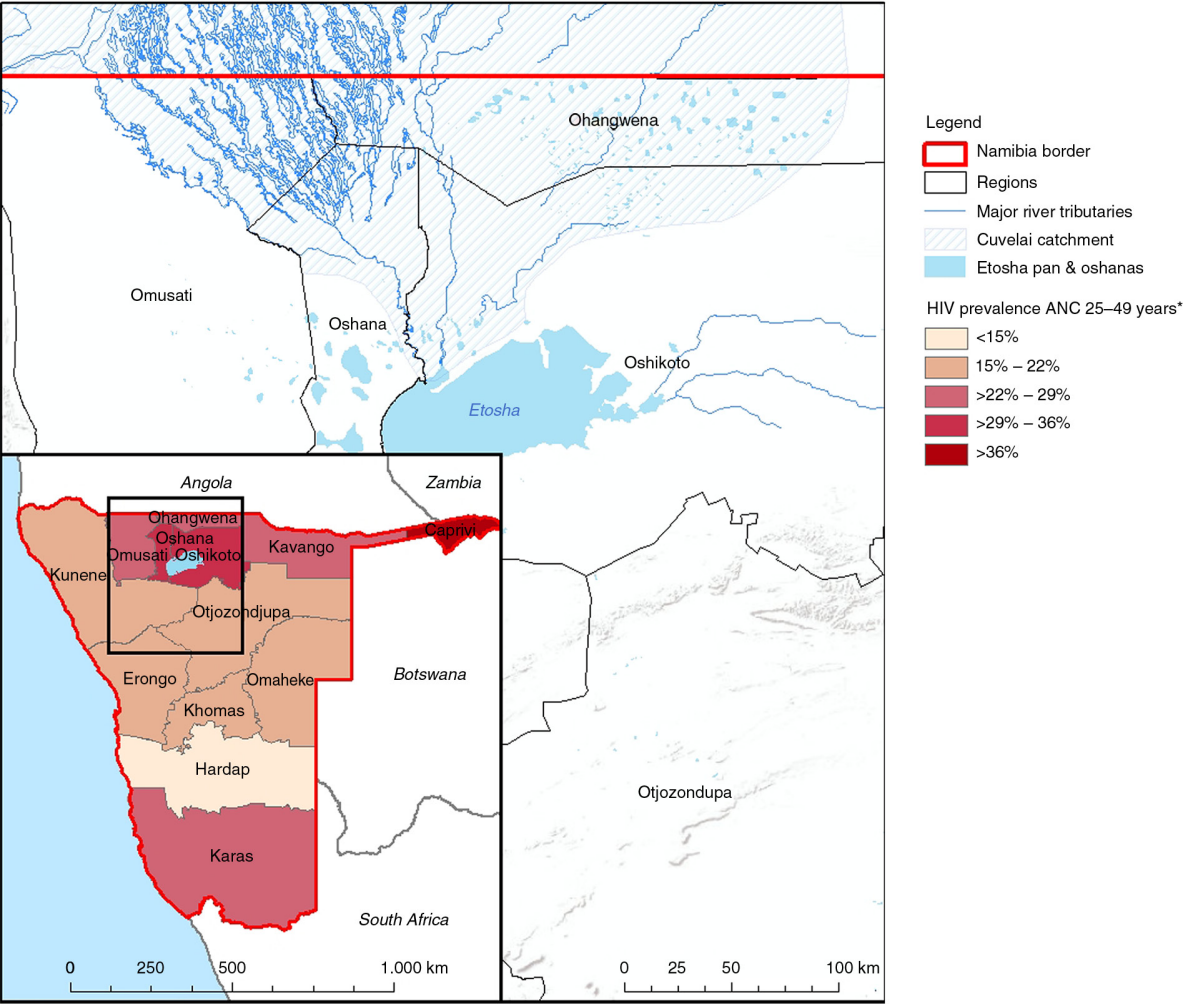
Map of Cuvelai catchment in the North of Namibia and the HIV prevalence among ANC attendees aged 25–49, median value from 2008. Data source: Directorate of Special Programmes, MoHSS Namibia (4). Map design: Institute for Hygiene and Public Health, Bonn, 2014.

In Ohangwena, most people live in rural areas and rely heavily on agriculture. The region suffers from many problems, including widespread poverty, unemployment, poor access to clean water and sanitation, weak infrastructure, and limited access to health facilities and services. The region is part of the Cuvelai-Etosha basin and the Okavango basin, situated along a flood plain. The climate can be described as semi-arid with high temperatures and rainfall varies greatly in amount and timing. The average rainfall per year is 480–600 mm, with the majority of rain falling from November to April (5). This means flooding and drought tend to be seasonal in Ohangwena. In recent years, heavy rains in the Cunene, Cuvelai, Okavango, and Zambezi river basins have caused widespread flooding and have particularly affected Ohangwena, as well as Caprivi, Kavango, Omusati, Oshana, and Oshikoto (6), where these disasters have had an additional negative impact on the Namibian health system (5–7).

According to the International Federation of the Red Cross and Red Crescent Societies (IFRC) (8), *a disaster is a sudden, calamitous event that seriously disrupts the functioning of a community or society and causes human, material, and economic or environmental losses that exceed the community's or society's ability to cope using its own resources. A disaster occurs when a hazard impacts on vulnerable people. The combination of hazards, vulnerability and low capacity to respond increases the disaster risk*.

In Namibia, floods – *Efundja* in the local Oshivambo language – can affect thousands of people, damage roads and vital infrastructure, leave health facilities and health outreach points inaccessible, and force many people to relocate (9).

Floods are a disaster situation for affected populations. The most likely to suffer from a disaster are the vulnerable groups within a community. These are children, orphans, pregnant women, nursing mothers, and people with chronic diseases. They are in need of functioning health care, appropriate hygiene, and a supply of healthy food. Moreover, they critically depend on their social care and support networks. After flooding, the delivery of health services and food is hindered, particularly in poor and isolated communities. Floods increase the high levels of poverty even further, and lead to food shortages as well as to reduced access to clean water and an increased spread of diseases.

The 2009 floods were devastating in northern Namibia and hit more than 700,000 people, displaced almost 55,000 people, and forced about 28,000 people into relocation camps.^1^ A total of 92 flood-related deaths were reported. More than 34,600 people received assistance from the Namibian Red Cross (10), many more were unable to access help. The floods in 2011 affected nearly 138,300 people in northern Namibia, of whom about 60,000 people lost their homes and approximately 18,000 moved to relocation camps. Communities were cutoff from health clinics, schools, and other social amenities. A total of 41 health facilities and 179 outreach health points were rendered inaccessible. In Ohangwena, 30 health facilities were damaged, four clinics were cutoff completely, so were 50 outreach points. Almost 60 schools were closed, affecting almost 16,000 pupils. More than 5,000 fields were destroyed and a total of 10 roads were damaged (9).

The high prevalence of HIV and extensive flooding are both threats in the Ohangwena region. This study aims at providing a deeper understanding of the impact that flooding has on people living with HIV (PLWHIV) as well as on HIV service providers in the Ohangwena region, Namibia. It takes a closer look at interlinkages between flooding and HIV focusing on the risks and threats caused by flooding for PLWHIV in the region. It also analyses the effects of flooding on HIV service provision^2^ and thus supports planning for emergency response and disaster preparedness in a context of high HIV prevalence.

## Design

The research was conducted within the Multi-sectoral HIV and AIDS Response Programme of the Ministry of Health and Social Services (MoHSS) of Namibia, supported by the Deutsche Gesellschaft für Internationale Zusammenarbeit (GIZ). It was carried out in close collaboration with the chief medical officer (CMO) of the Ohangwena region, and the programme officer for special programmes of the Ohangwena Regional Health Directorate and the Engela Health District. Data were collected from March to May 2012 in Ohangwena region and in the two constituencies in the region most affected by floods (Endola and Ohangwena – see Fig. 1). The data collection was carried out at different levels. At the local level, PLWHIV affected by flooding were addressed by semi-structured interviews in Engela health district; at regional level, the views of representatives of various line ministries,^3^ public sector institutions, and civil society organisations were gathered through expert interviews and focus group discussions; at national level, a feedback meeting included representatives of Namibian ministries, international governmental and non-governmental organisations, and other institutions and stakeholders (see Table 1).

**Table 1:** Overview of all research partners and methods

**Engela health district**	
Semi-structured interviews in Endola constituency with four women LWHIV representing four affected families from	Semi-structured interviews in Ohangwena constituency with three women LWHIV representing four affected families from
Ondjabalala VI, Ondjadjaxwi, Ohalushu, Okahenge	Ohangwena, Ohangwena – 2, Ohangwena YaAmon
**Ohangwena region**	
Expert interviews with	
Health authorities, including regional primary health care supervisor and registered nurse from Eenhana district	
Emergency management authorities, including head of Regional Emergency Management Unit and head of Red Cross Regional Disaster Management	
Other institutions: Solidarity Community Care Organisation	
Focus group discussions with	
Public sector institutions including:	
Regional Health Directorate (chief medical officer, programme officer of special programmes), regional representatives of ministries (education, gender, agriculture), member of the regional council, police officer, health care worker	
Civil society organisations including:	
Volunteers of local NGO total control of the epidemic (TCE), Red Cross volunteer, support group members, pastors of churches in Endola and Ohangwena constituencies	
**National level feedback meeting held in Windhoek on 16 May 2012**	
Governmental institution participants:	
Directors of special programmes, logistics, epidemiology of the Ministry of Health and Social Services, Namibia	
Representatives of Ohangwena Health Directorate and Ohangwena Regional Council Emergency Management	
Representative of the office of the prime minister, representatives of the ministries of: agriculture, education, works & transport, gender equality & child welfare, safety & security, roads authority, defense	
Representative of the National Planning Commission	
**International organisation/government organisation participants:** World Health Organization, Joint United Nations Programme on HIV/AIDS, United Nations Development Programme, United Nations Children's Fund, United Nations Population Fund, Gesellschaft für Internationale Zusammenarbeit, United States Agency for International Development	
**Non-government organizations:** Namibian Red Cross, National Association of Chiefs of Police, Positive Vibes, Legal Assistance Centre, Namibia Networks of AIDS Service Organisations, SAfAIDS, Namibia NGO Forum	

### Qualitative approach

For this study, a qualitative approach was chosen and grounded theory was applied. The analyst gathered data openly before and while analysing it by applying different theoretical frameworks and concepts, thus moving from the data to an emerging set of concepts in order to develop a theory. An exploratory study design was used to describe the situation and magnitude of the problem. Because of the complexity of the research, a mix of various methods was used (see Table 1). The main research questions that evolved during the research process were: What was the impact of recent floods on PLWHIV and their health in the Ohangwena region, and how have the disaster situations affected their access to health care and HIV services? How have service providers reacted to flooding and which mechanisms of disaster risk management are in place?

### Methods and data collection

The target population for the study was flood-affected PLWHIV in the two most flood-prone constituencies, Endola and Ohangwena, in the Ohangwena region. These people belong to a poor rural population, depending on natural resources and subsistence farming for their livelihood. Assisted by the NGO Total Control of the Epidemic (TCE),^4^ which maintains a record on the support activities for all PLWHIV in the region, PLWHIV compromised by flooding in these constituencies (see Table 1) were identified through their HIV support groups. Seven women living with HIV who were severely hit by floods were chosen by the snowball method. They participated in semi-structured problem-oriented interviews that followed key questions, after having given their free and informed consent^5^ to contribute to the study. The questions addressed the consequences of flooding on the lives, health, nutrition and behaviour of PLWHIV and investigated the general health and access to HIV services. The interviews took 45–90 min each and were all conducted by one of the researchers and her trained partner, a registered nurse from Eenhana district. During the interviews held in Oshivambo, all answers were simultaneously translated into English. The interviews were audio-recorded or, where the participants refused this, extensive notes were taken. No male respondents were recruited for the interviews, as they did not take part in TCE support group activities and if available, were not interested in taking part in the study.

Different stakeholders in HIV management and emergency management were chosen to participate in two focus group discussions. They were selected as a representative set of people involved in disaster risk management during flooding. One group contained nine members of civil society organisations while the other group consisted of seven representatives from public sector institutions (see Table 1). Their professions represented disciplines and topics relevant to the research questions, including health care, education, agriculture, gender, and safety. Everyone had the chance to share their ideas, knowledge, and recommendations, and to contribute their views on key questions in a time period of 90–120 min.

Five key stakeholders from Ohangwena region were selected to be interviewed as a supplementary source of information, as they worked in health care and HIV service provision and emergency management. They were identified after consultation with the Regional Aids Co-ordinating Committee (RACOCC) and the Emergency Management Unit of Ohangwena. Participants included a disaster risk management officer, a representative from the Emergency Management Unit, a primary health care supervisor, the chairperson of an NGO providing HIV services, and a representative of the Red Cross. The interviews were structured around the unique tasks and functions of the interviewees and took 30–120 min each. The focus was on thematic probing, while comparability, completeness, and generalisability were checked as well.

In addition, a national feedback meeting in Windhoek took place, where the preliminary research results were presented and discussed. It was addressed to stakeholders in the field of HIV and flooding (Table 1) and helped to gain further input for a critical reflection on the study outcomes.

### Data analysis

After the data collection phase, the audio-recorded material was transcribed by applying F4 software. To prevent misinterpretation of the recordings, the analyst checked them against the original transcript, listened to the original audio recordings again, and discussed them with her research partner. The data were analysed in an interpretative hermeneutical way following grounded theory (11). The process of data collection was determined by the theoretical frameworks and concepts on livelihoods, natural hazard research, and health behaviour, which were chosen and applied based on the findings. The *livelihoods framework* by DFID addresses the access to livelihood assets, including human, natural, financial, physical, and social capital, which depends on the scope of action. It is determined by the exposition towards a shock, trends, and seasonality, and by transforming structures. The more assets an actor can access, the slighter is its vulnerability and the bigger are his capacities of coping with risks, shocks, and stress during crisis. Following this model, there are two causes for a crisis: the lack of resources and the lack of safety strategies. According to the approach of *natural hazards research*, exposure is determined by proximity to the source of the threat, incident frequency or probability, magnitude, duration, or spatial impact. Social impact and response are measured by threats to lifelines or infrastructure to support basic needs, special needs populations, poverty or wealth indicators, gender, race, and others. There is a high risk of disaster when one or more natural hazards occur in a vulnerable situation. The risk of a disaster is a compound function of the natural hazard and the number of people with varying degrees of vulnerability, who occupy the space and time of exposure to the hazard event (12). Health behaviour and the process of behaviour change is according to BANDURA, not driven by inner forces, but by external factors. It describes human behaviour in terms of a model of reciprocal determinism in which behaviour, cognitive and other personal factors, and environmental events all interact. In contrast, the theory of planned behaviour distinguishes between behavioural intention and actual behaviour. It suggests that behaviour depends on one's intention to perform certain acts, which is, however, influenced by external factors preventing or supporting an individual to engage in certain behaviour.

A central part of the data analysis was the interpretation of the data by coding with MAXQDA© software. Different hierarchical types of coding (namely open, theoretical, and selective) were applied, following the ‘theoretical sampling’. Methodological transparency was very important in the data analysis. To prevent misinterpretation, categories were predefined in as minimal a way as possible which was also consistent with the need for reliability, dependability, generalisability, and organisation of the data. To validate the data gathered by different methods, the findings from the interviews with PLWHIV were triangulated with information from the discussions, the expert interviews, and the feedback meeting. As literature on HIV and flooding *per se* was lacking, the findings were cross-checked for further validation with a literature review on a) HIV and emergencies, b) HIV and environment, and c) flooding and health/disease.

## Results

The empirical results reveal that HIV and AIDS and flooding interact in three important ways: 1) the impact of flooding on PLWHIV, 2) the impact of flooding on HIV service provision, and 3) the impact of flooding on the transmission of HIV. Furthermore, the study gives insights into 4) the mechanisms of public health disaster risk management in the Ohangwena region.

### The impact of flooding on PLWHIV

PLWHIV reported their everyday lives, their livelihoods, and their work to be severely affected by flooding and its consequences for houses, fields, livestock, assets, income, and increased expenditure.

The people living with HIV and AIDS, their crop and Mahangu field used to be washed away by flood. (HBC, Ohangwena region)

The destruction of infrastructure forces PLWHIV to stay at home or to relocate to relatives’ place or relocation camps and makes transport difficult or even impossible. During and after flooding, PLWHIV face food insecurity, which, together with decreased quality and accessibility of water, sanitation and hygiene (WASH), can lead to a deterioration of their health status.

I don't feel well during flooding. My body is going down and weakening during flooding. (PLWHIV, Ohaluxjo)

Immune resistance (…) gets less when you are HIV positive. So you need to eat good and enough food. (Red Cross, Ohangwena region)

Diseases like malnutrition, malaria, diarrhoea, acute respiratory infections, and mental problems can be more prevalent during flooding.

There are definitely more diseases, many people get sick with malaria, cholera, diarrhoea and flu. (PLWHIV, Ohangwena 2)

PLWHIV (…) do not only experience HIV, but also waterborne diseases. (HBC, Ohangwena region)

All of these aspects also apply to those affected by the flood who are not HIV-positive, but the impact is bigger on PLWHIV and people with other chronic diseases as they depend on their social networks.

The social life is disturbed somehow. (Pastor, Ohangwena)

During flooding, numerous churches, schools, and community centres remain inaccessible. This hinders quite a number of PLWHIV from practicing their social activities, exchanging information on HIV and AIDS, and receiving advice and assistance from friends and acquaintances.

The church is also affected (…) and those people (…) won't be able to come to the church. And then if there is nobody (…) who is giving information on HIV/AIDS, those people are left behind. (Pastor, Ohangwena constituency)

During flooding, some PLWHIV fail to take their ART medication due to limited accessibility and the fear of stigmatisation and discrimination, particularly in relocation camps. In such a setting, little privacy and confidentiality can be guaranteed and people's HIV status cannot be kept secret.

When people are in camps, there is no privacy (…). People are scared to take it [their medication] because they don't want to be seen. (Pastor, Endola constituency)

### The impact of flooding on HIV service provision

According to the PLWHIV and HIV service providers interviewed, HIV service provision is negatively impacted by flooding. The destruction of infrastructure presents a huge barrier for the delivery of prevention, treatment, care and support services. Some of the PLWHIV are unable to reach health facilities or outreach points and remain without sufficient assistance or services until the waters recede.

Crossing the water is dangerous, so you sometimes don't want to do that. Some [PLWHIV] have defaulted their service, they are no more coming to get their ARV treatment for fear or phobia of water. (PLWHIV, Ohangwena)

Transport is a critical issue for several PLWHIV and for the service providers, as vehicles or transport animals are lacking and expensive and as health facilities are only accessible on a limited scale.

When it is flood, it is difficult for someone, who falls sick, to go to the hospital. (Programme Officer for Special Programmes of the MoHSS, Ohangwena region)

Many clinics are overcrowded and people have to wait hours to consult a doctor. Due to flood-related injuries and infections that need to be treated, the health facilities’ capacities are overstretched. Some clinics are even out of order.

Sometimes even the clinics are flooded and do not operate. They are not accessible, so they [PLWHIV] are no more coming to get their ARV treatment. (Nurse, Eenhana district)

Although these effects may apply to the whole healthcare sector during a flood, the impact is particularly great on PWLHIV.

Access to antiretroviral (ARV) treatment is disrupted, causing more defaulters during a flood. Many PLWHIV miss the dates for collecting their medication because of the flood water and the widespread inaccessibility of health services and facilities.

One woman had to cross a very deep stream (…) to go to the health facility (…). Her ARV finished and she didn't want to threaten her health. So she risked her life to get her treatment. (Solidarity Community Care Organization (SCCO), Ohangwena region)

Crossing the water is dangerous, so you sometimes don't want to do that. Some have defaulted their services for fear of water. (PLWHIV, Ohangwena)

As access to medication becomes scarce, some PLWHIV share their medicines with others and therefore do not take the necessary dose themselves. During flooding it is not possible to trace and reach defaulters in the villages.

Some PLWHIV do not take their medication regularly and default because of floods and so much rain (…). (PLWHIV, Ohangwena 2)

PLWHIV, most of them are defaulting (…) because they don't have access to hospitals to get medication. (PHC Supervisor, Engela district)

Different representatives of NGOs providing home-based counselling and care^6^ said it is nearly impossible to maintain their work during flooding.

It doesn't help for someone who needs ARV if he or she is not counselled. If there is no counselling, there is also no way to check if people take their medication. (HBC, Ohangwena constituency)

The availability of health care workers decreases due to work overload resulting from additional outbreaks of disease^7^ accompanying flooding. There are not enough staff to both continue working in clinics and health centres and also serve in outreach and relocation sites. Even if extra services are brought to the PLWHIV in some of the flooded villages, for example, by helicopter, it is not possible to serve all people and all sectors in all locations.

Logistically, you cannot reach everybody, even the limited services you want to provide the people with. It is difficult. (Programme Officer for Special Programmes of the MoHSS, Ohangwena region)

The quality of care and support services is diminished for a long period of time, and the work is multiplied during flooding, for example, due to the relocation camps.

In the process of flooding our other activities get affected. Our staff are really stressed to the limit. They are overworked and the quality of services is poor (…). And at the end of the day the quality of health care diminishes. (CMO, Ohangwena region)

The general management of service providers is also affected, as regular schedules of meetings are disrupted by immediate actions and ad hoc emergency meetings. This has a strong negative impact on HIV services and their monitoring.

The floods affect our existing plans. You get 3 to 4 months where we are completely losing focus. We all concentrate on the floods and we forget about our main plans, our main monitoring and evaluation exercise, which includes even the HIV and AIDS monitoring and evaluation. (CMO, Ohangwena region).

As well as HIV, all other activities are badly affected, including outreach services, tuberculosis (TB), and vaccination programmes. Moreover, the service providers, especially the MoHSS, report a huge financial impact in terms of an increased use of medicine and pharmaceuticals, overtime, transport, and other issues. Supplies need to be bought, as there are no supplies and stocks for any kind of emergency or outbreak.

Challenges for HIV service providers during flooding are: poor communication and poor dissemination of information between local, regional, and national levels; the administrative procedures the stakeholders have to follow; too little involvement of the affected population; and aid sometimes being poorly targeted (CMO, Ohangwena region).

### The impact of flooding on the transmission of HIV

The risk of HIV transmission increases during flooding because of behavioural changes and environmental circumstances. PLWHIV may use fewer preventive measures because of decreased social control and a temporary acceptance of higher risks. Information on HIV and family planning is limited and condoms in particular are not accessible to many people, who therefore practice unsafe sex.

When people are flooded in the community, condom distribution is not possible anymore, putting people at a higher risk of communicating HIV, as well as sexually transmitted diseases. (HBC, Ohangwena constituency)

Fewer PLWHIV receive counselling and psychosocial care during floods. It is difficult for HIV service providers to undertake HIV testing, so the virus is transmitted by people who are unaware they have HIV. This is particularly problematic when people are displaced, families are separated, or people have multiple sex partners.

Another factor which affects the risk of HIV transmission is the increase in physical and sexual violence during flooding, which is linked to high consumption of alcohol, limited control, and diminished safety and security.

Crimes increase during floods; even sexual violence happened. (PLWHIV, Ohaluxjo)

And there is the issue of alcoholism. (Nurse, Eenhana district)

People – mainly women and orphans and vulnerable children (OVC) – have to walk long distances to get medication, food, clean water, and firewood, and, especially at night, may be exposed to violence and rape. This was reported by the people interviewed. The same may happen in relocation camps.

People are getting infected with HIV during flooding because they are raped. The environment is not conducive, there are no lights in the relocation camps and people have to walk long distances. (Red Cross, Ohangwena region)

We have difficulties to protect the people (…). We need to protect the houses, the relocation sites, (…) it is nearly impossible. (Representative of Namibian Police Force (NAMPOL), Ohangwena region)

OVCs are reported to be the most vulnerable. They do not have secure livelihoods or parents and family to take care of and protect them, and they have little in the way of social and security networks to support them. They are the first ones to drop ARV treatment and default.

Finally, malnutrition and disease weaken the immune systems and therefore increase the likeliness of virus transmission.

When there is just no food, they have weaker bodies. (PLWHIV, Ohangwena)

**Fig. 2:**
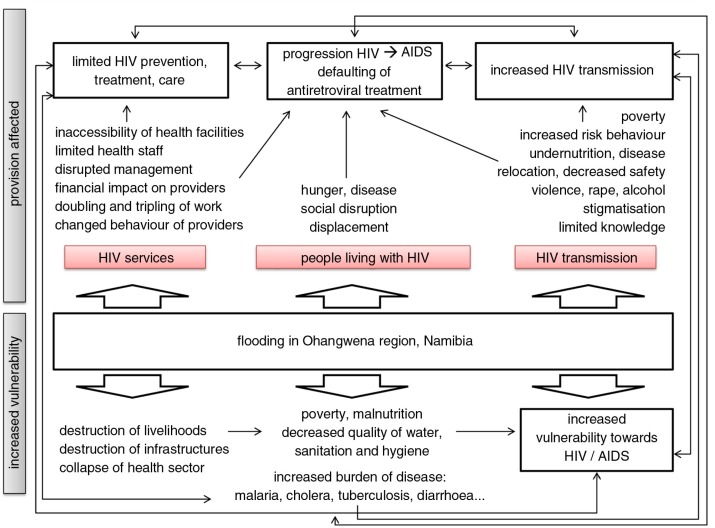
HIV & flooding framework.

The most important results of the impact of flooding on PLWHIV 1), of the impact of flooding on HIV services 2), and of the impact of flooding on the transmission of HIV 3) are displayed in the *HIV & Flooding Framework* (Fig. 2). They represent the interlinkages between HIV and flooding, lead to an improved understanding of this complex issue, and provide the basis for disaster preparedness.

### The mechanisms of public health disaster risk management in the Ohangwena region

The Namibian government has a national disaster plan in case of floods. In each region, a permanent Regional Disaster Risk Management Committee is responsible for coordinating all the disaster-related activities and stakeholders in the region. It is spearheaded by the regional council, and all line ministries and NGOs are members of the committee. Each sector and line ministry further has their own plan; they do not rely solely on the structure at the regional level (CMO, Ohangwena region). When a disaster occurs, the regional council calls all stakeholders together, they have emergency meetings and are then given responsibilities and an action plan (Representative of the NRC).

When it comes to a flood, it is like an ‘Automatic Call’ touching and involving everybody. (Representative of NAMPOL, Ohangwena region)

There is close collaboration between all units involved and substantial amounts of resources are set aside for emergency response. When the floods start, relocation sites are set up and the regional council and the Red Cross determines which services need to be made available: schools, hospitals, units for police, and others. The Ministry of Defense provides boats and helicopters, as well as funds to build temporary bridges to access difficult to reach areas. The regional council together with the Ministry of Agriculture identifies the farmers and people most affected by flooding to give them food aid (Representative of the Ministry of Agriculture, Ohangwena region). At the health sector level, preparedness for disasters, especially disease and outbreak prevention has a high priority. Therefore, expanded programmes of immunisation and immunisation campaigns are promoted and organised by the MoHSS. Health care workers and volunteers are trained by the Namibian Red Cross so that they are ready to be deployed in the affected areas. Training is also organised at constituency level and village level, so that people are in a better position to respond (representative of the NRC). Water purification is provided by the Namibian Red Cross. Furthermore, there are volunteers trained by the Ministry of Education to take care of children in the relocation camps and there is even camp management training (representative of the Ministry of Gender, Ohangwena region). All activities are coordinated through the Regional Disaster Risk Management Committee, and all stakeholders pool their resources, so that there is no duplication of activities. All interviewees agreed that during flooding all stakeholders respond very well. According to the public and civil society representatives, there is a well-coordinated response of all sectors during a flood, whereas collaboration in normal circumstances needs improvement.

All of the sudden when we are facing emergencies, we start working nicely and we meet so regularly and we exchange our information. These are positive impacts that we can get from the flood. (CMO, Ohangwena region)

… they [the government, civil society and other stakeholders working with HIV] are all trying their level best to support the PLWHIV during flooding, to reach those affected, to provide all kinds of HIV services and especially supervise ARV intake. (CMO, Ohangwena region)

Disaster response includes HIV service provision and ARV treatment (ART). This means that despite the problems reported earlier, some PLWHIV do receive additional care during floods. The RACOCC provides information and supports HIV service providers. Up to 6 months’ worth of ART is provided to patients when flooding starts, so that they need not access a hospital or a health facility. A special registration system for ARVs is set up and information is sent to the MoHSS. Hospitals communicate with the ART clinics, doctors, and health care workers, trying to make sure that patients receive their treatment. Some PLWHIV are transferred to closer health centres to access medication more easily. On the local level, PLWHIV are advised by NGOs, home-based care organisations, health care workers, and others on how best to behave during flooding. HIV service providers deliver health-related information on the radio, via pastors and school principals, or advise community health workers.

## Discussion

The case of Ohangwena shows that PLWHIV are very vulnerable and face additional hardship during flooding (Table 2). PLWHIV's behaviour becomes more risky, and their vulnerability increases even more, leaving them with an exhausted capacity to cope. Okal and Bergman (13) add that, if PLWHIV are unable to recover adequately from emergencies like flooding, they become weak and unproductive and may slip into a permanent state of crisis in the long run.

**Table 2:** Overview of empirical data aggregation and analysis

			Empirical findings				Literature	
			
Category	Code	Subcode	PLWHIV	FGD	Experts	Feedback	UNAIDS	General
Impact on PLWHIV	Health status	Decreased immune system	x	x				x
		Additional diseases and malnutrition	x	x	x	x	x	x
		Decreased effect of ART	x	x	x	x		x
		Defaulting, interrupting of ART	x	x	x	x	x	x
	Social network	Disruption of families and social network	x	x	x		x	x
		Destruction of schools and churches	x	x	x	x	x
		Stigma and health-seeking behaviour	x	x	x	x	x
Impact on HIV service provision	Inaccessibility of HIV services	Distance to health facilities	x	x	x			x
		Destruction of infrastructure	x	x	x		x	x
		Increased cost of transport	x	x	x		x	x
		Inaccessible health facilities	x	x	x	x	x	x
		Disruption of ART adherence	x	x	x	x	x	x
		Inaccessible schools and churches	x	x	x		x	x
		Limited staff	x	x	x			x
		Limited health sector resources		x	x			x
	Barriers to using HIV services	Stigma, disruption of social networks	x	x	x	x		x
		Food shortage and lack of safe water	x	x	x			x
	Positive impact on service provision	Increased service provision		x	x			x
		Provision by helicopters, etc.	x	x	x			x
Impact on HIV transmission	Inaccessibility of services	Lack of information	x	x	x	x	x	x
		Lack of condoms and family planning	x	x	x		x	x
		Lack of HIV testing, PMTCT, and ART	x				x	x
	Barriers to effectiveness of services	Counselling and care	x	x	x		x	x
		Malnutrition	x	x	x	x	x	x
		Defaulting ART	x	x	x	x	x	x
	Factors influencing transmission	Decreased safety and camp conditions	x	x	x	x		x
		Sexual violence, crimes, alcohol abuse	x	x	x	x	x	x
		Unsafe sex		x	x			x
		Transactional and survival sex					x	x
		Disruption of families and social networks	x	x	x			x
		Outbreak of diseases	x	x	x		x	x
		Decreased immune system	x	x				x
		General risk behaviour		x	x		x	x
		Stigmatisation		x	x	x		x

### The impact of flooding on people living with HIV

PLWHIV need a good nutritious diet and have higher energy requirements than the non-infected to lead a healthy life. Therefore, flood-related crop failure and food insecurity leading to malnutrition hit them hard. HIV is more virulent in malnourished people, and HIV-induced immune impairment and the higher risk of subsequent infection can worsen their nutritional status further (14). PLWHIV's burden of disease is further strained by the intake of contaminated water and a diminished quality of WASH which can result in an increased occurrence of water-related and vector-borne diseases (12, 15). Malaria, cholera, diarrhoea, other flood-related diseases, and TB, which increase with crowding and poor hygiene, are likely to spread (16), especially in relocation camps. UNAIDS (17) reported that almost 80% of the flood-affected populations in Namibia's North named diarrhoea as their main health problem during floods, followed by 67% naming malaria, 60% respiratory tract infections, 53% opportunistic diseases in relation to HIV, and about 30% TB. According to Laufer and Plowe (18) and UNEP & UNAIDS (19), a harmful interaction between malaria and HIV exists: while an HIV infection worsens the progress of malaria, an untreated malaria infection can increase the HIV viral load. Generally, a weakening immune system due to a lack of adequate nutrition aggravates HIV infection and hastens the progression from HIV to AIDS, while at the same time decreasing the effectiveness of ART medication (13).

Another important aspect to be mentioned is the impact that flooding has on PLWHIV's social support system. If they lose their family members, social networks, and community institutions due to flooding, PLWHIV's vulnerability is strained further. According to UNAIDS (17), 87% of the PLWHIV interviewed reported a disruption of educational services, thereby limiting vital knowledge transfer about behaviour. The importance of churches and pastors in dissemination of HIV-related information in the Ohangwena region needs to be emphasised, as it was mentioned by all research participants. PLWHIV and experts agreed that there was a reduction in use of HIV services among relocated PLWHIV due to social disruption and fear of stigma, which is consistent with the outcomes of the literature review. HIV transmission can increase as a result of people not seeking health care. This connection was identified by experts working with HIV in Ohangwena and is underpinned by Crezpaz and Marks (20).

In addition, PLWHIV can face mental health consequences due to a *loss of place* caused by flooding. It was reported by the majority of the women interviewed in Ohangwena that the loss of goods and of their homes as well as relocation due to flooding, resulted in an additional burden for the affected population. This is in line with Few (21), WHO (22), and IASC (23).

### The impact of flooding on HIV service provision

PLWHIV face insecure access to basic health services due to inaccessibility and the destruction of infrastructure. Although treatment is usually free, transport is normally expensive and prices rise during flooding (17), so PLWHIV experience difficulties reaching the health facilities to receive their free health services. These external factors prevent PLWHIV in seeking health-supporting services and change their behaviour. All the women interviewed and some experts and participants in the focus group discussions reported that access to HIV services had been severely hit in recent years, as flooding disrupted services and supplies in the region. No figures on the adherence to HIV services were available and no official data could be provided by the Regional Health Directorate. The representatives working on HIV and health service provision reported the general health sector to be severely affected by flooding. This leads to a lower priority of HIV compared to the immediate health threats and other needs such as food aid, shelter, and safe water. The UNAIDS study (17) agrees by reporting the destruction of outreach services, of support for OVC, and of condom distribution, as well as the total disruption of home-based care and counselling services, which are of particular concern for those cutoff by flood waters. Simon (24) states that HIV in emergencies has been ignored for years, because it is not seen as a life-threatening condition. The IASC guidelines on the inclusion of HIV, as well as the guidelines in the Sphere Handbook, address this problem and try to fill this gap (25, 26). However, little implementation has taken place so far. This is of particular concern as the HIV situation in Namibia and in other countries or communities with high HIV prevalence is a disaster situation *per se*.

The inaccessibility of health services caused by a disaster such as flooding can negatively influence health-seeking behaviour and therefore adherence to ART. According to UNAIDS, access to ART was disrupted severely in the northern regions during flooding, with 23% of the people interviewed missing their medication and the Ohangwena region containing the highest proportion of defaulters. Okal and Bergmann (13), on the contrary, found that ART supplies are not disrupted as much as often feared. Samuels et al. (14) even talk about emergencies being an entry point for HIV service provision, as in some places defaulter tracing is installed to reach people who need medication.^8^ PLWHIV themselves did not mention the positive side effect of increased service provision during flooding but stressed their hardship in accessing ART. This contradiction should be investigated further, as statements on the access to HIV services differ widely between the people in need and those who provide support. Regardless of all the obstacles, many PLWHIV perceive that the positive outcomes of maintaining ART and HIV services outweigh the barriers, and therefore try to stay enrolled in their medication programme. This is of course not possible for all PLWHIV, as the main factors affecting adherence to ART and other health services are the inaccessibility of health facilities, the lack of funds to cover transport costs to health facilities, the lack of food and clean water (15), inadequate knowledge, and the lack of social support systems. These problems are addressed in the standard on essential health services in the *Sphere Handbook* (26), which specifies that access to information, education, and communication (IEC); condoms; and ART must be safeguarded generally, especially in countries that show a high HIV prevalence.

### The impact of flooding on the transmission of HIV

A reduced use of HIV services, including opportunistic infections, and a decline in psychosocial support compared to the normal situation were reported by the experts interviewed in Ohangwena. Increased risk behaviour by PLWHIV as well as other people enhances vulnerability towards HIV. Factors contributing to the transmission of HIV include the lack of livelihood assets such as resources and food, external drivers caused by the natural hazard such as the inaccessibility of infrastructure and HIV services, unsafe camp conditions, and an increase in transactional sex as one very risky way to cope with the disaster. Relocation camp conditions expose populations further to the risk of contracting HIV or other diseases such as TB by increased proximity to others, unsafe environments, and dangerous conditions resulting in rape and violence. PLWHIV as well as all stakeholders interviewed agreed on this point. The separation of families plays an important role, prompting people to have extramarital intercourse and even children to engage in sex. UNAIDS (17) found that 25% of the OVC interviewed in their study lacked adult guardian care during flooding. This puts them at a higher health risk, along with lack of shelter and lack of adequate food. Women live under precarious economic circumstances and many prostitute themselves for food, money, safe water, or other vital goods (28). It is not clear how men can afford to bargain for sex, as money is scarce for them during flooding as well. Interestingly, such ‘survival sex’ was not mentioned at all by any of the women in Ohangwena and only in an indirect way by the people working with HIV in the region. This might be because of restraints or unpleasantness in addressing this issue. Risk behaviour was mentioned with regard to HIV transmission but was not explained further by the respondents. Increased consumption of alcohol leads to risky behaviour and violence, (20) which increases the spread of HIV significantly (26).

### Disaster risk management in Ohangwena region

According to the key informants, during extreme flood situations, collaboration and coordination between stakeholders improves compared to the rest of the year. They reported knowing their roles and responsibilities during a disaster and committing themselves to work together and to discuss which actions have been taken previously and how they can be improved. Simons (24) disagrees and mentions limited cooperation among different stakeholders and humanitarian actors during emergencies. According to him, humanitarian and HIV-related activities often take place on parallel tracks rather than being planned and implemented in a coherent way. According to some health professionals in the region and in line with the regional health records and findings from the literature (14), another positive consequence of flooding can be that HIV service provision is improved. This does not however mean that everyone in need can profit from the increased supply.

Based on the findings of this study and by applying the *HIV and Flooding Framework* (Fig. 2), and also by considering experiences from other emergency situations, disaster preparedness in northern Namibia could be improved. One cornerstone of disaster preparedness efforts is the identification of responsibilities, communication lines, and coordination structures to improve efficiency and coherence of emergency response activities. This study identified some of those and could therefore have an added value. Another important step with regard to disaster preparedness would be to conduct regional or local vulnerability and capacity analyses to identify those individuals, families, or groups most at risk in a disaster situation and to identify available resources.

### Limitations of the study

The research topic gives rise to limitations with respect to both literature and data. So far, little research has been carried out on the impact of flooding on PLWHIV. The exact number of people without access to HIV prevention, treatment, and care is difficult to assess as not enough surveillance is done during floods, and no concrete data were available because the lack of infrastructure, lack of staff, and limited financial resources hinder monitoring and evaluation of the health situation. The research sample at the local level was quite small: only seven female PLWHIV from the rural poor population were interviewed. The researchers were not able to interview any male respondents – either they were not interested or none were available. As no men were included in the study, the interviewees’ answers cannot be generalised and do not show gender-based differences. Triangulating the women's statements with those of the experts, which included experiences with male PLWHIV and PLWHIV from differing social, cultural, and economic backgrounds helped to fill the research gap. The choice of the experts for the interviews and focus group discussions may also have influenced the outcomes of the study. We sought to minimise possible bias by choosing a wide range of different stakeholders from local, regional, and national levels, and by using a large number and variety of methods.

## Conclusions and recommendations

**Fig. 3:**
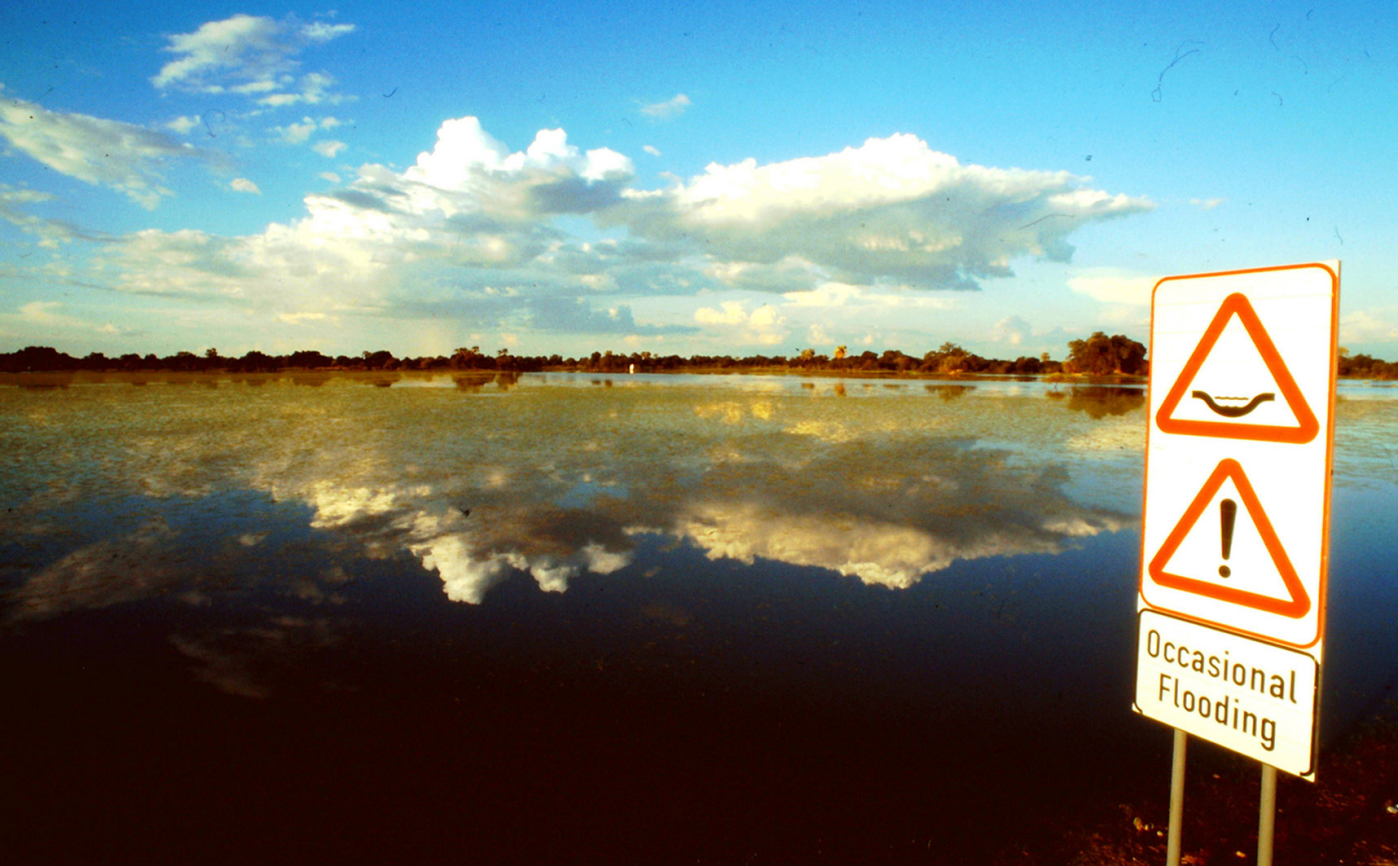
Annual floods in the Ohangwena region pose major problems to everyone affected.

The study and the *HIV and Flooding Framework* (Fig. 2) developed in the course of this research revealed that *Efundja* – as the annual floods are called – pose major problems to everyone affected (Fig. 3). Everyday lives, livelihoods, work and income, as well as homes and living conditions are impacted adversely by such events, which force many individuals and families to temporarily move to relocation camps. In these camps, malaria; cholera; diarrhoea; and other flood-related diseases, such as malnutrition, mental problems, and TB, are more prevalent during flooding. PLWHIV in Ohangwena are more vulnerable in the case of flooding and the subsequent health problems than people not affected by HIV. PLWHIV's immune systems and their health status are generally weak, thereby limiting their coping capacities. Flooding is a barrier for quite a number of PLWHIV in maintaining their social activities, exchanging information on HIV and AIDS, and receiving advice and assistance from friends and acquaintances. Furthermore, numerous PLWHIV default their ART medication due to the limited accessibility of health services and the fear of stigmatisation and discrimination, particularly in relocation camps. The negative impact of the same factors which threaten them under regular living conditions – poverty, food insecurity, unsafe WASH, little social capital, stigmatisation, limited access to health facilities, HIV services and ART, among others – is intensified in addition to pre-existing burden of HIV. Of particular concern is the fact that women and OVCs are the most affected, and are left without assistance, care, education, or guidance.

A dual risk of floods exists for the health of PLWHIV: the increased risk both of infection and disease due to the inaccessibility of health services and ART. This entails a health and economic threat to PLWHIV and HIV service providers in the Ohangwena region. This study demonstrates that vulnerabilities and health risks of PLWHIV will increase in a disaster situation such as flooding if access to HIV prevention, treatment, care and support are not addressed and ensured. The unpredictability of the timing, the increased frequency, and the extent of flooding pose additional challenges to health and the health system's response.

The results of this study are in line with the literature on the impact of emergencies on PLWHIV and general emergency standards (26). The situation in Ohangwena region is, however, further complicated by additional health problems affecting PLWHIV and creating pressure on the health sector. Health problems and communicable diseases which evolve over the course of a flood (and disasters in general) include malaria, diarrhoea, and respiratory diseases, as well as stress reactions and mental health disorders. These are a significant challenge to individuals who are already vulnerable as they are living with disabilities or are chronically ill from HIV and/or TB, and who are therefore more susceptible to these diseases. In addition, due to the widespread destruction of subsistence agriculture, the main source of income, flooding hits PLWHIV in the Ohangwena region particularly hard. The findings of this study and the *HIV and Flooding Framework* (Fig. 2) are not specific to Ohangwena and can be transferred to any flood-affected region, which has a high HIV prevalence and relies mainly on subsistence agriculture. They serve as a model case for other vulnerabilities related to health and health service provision under disaster conditions (29). The impact, however, will vary according to a number of factors: the physical and geographical characteristics of the area, the regional climate, the social and human resources in the region, the distribution of income, wealth and power, and the culture and behaviour of the people affected.

This study shows that disaster risk managers in the Ohangwena region have already developed a mechanism that addresses PLWHIV during flooding. Different sectors collaborate, share responsibilities, and improve their communication and disaster response with regard to HIV service provision. To reduce flood-related risks for PLWHIV, the authors make the following recommendations:

- Preactive identification of all stakeholders involved
- Preactive clarification of responsibilities, improved communication, and coordination of all sectors involved
- Inclusion of detailed capacity and vulnerability assessment of flood-affected populations in disaster response plans
- Application of lessons learned from previous floods to strengthen capacity and identify potential gaps
- Reduction of flood-related risks for PLWHIV and other chronically ill and vulnerable groups through integrated disaster preparedness measures for future floods
- Improvement of disaster preparedness by applying the *HIV and Flooding Framework* and by considering experiences from other emergency situations.

## Footnotes

1 Relocation camps are set up by the Namibian Red Cross during flooding to provide shelter, food, water, and other items to the victims.

2 HIV services include prevention, treatment, and care: voluntary counselling and testing, information and awareness activities, community counselling, home-based care (HBC), antiretroviral treatment (ART), support for orphans and vulnerable children, prevention of mother-to-child transmission (PMTCT), mobile services, and other activities.

3 A term used in Namibia to mean the ministry with direct responsibility for dealing with a situation.

4 TCE is a community mobilisation programme targeting HIV and AIDS in Namibia. It is implemented in partnership with the Global Fund through the MoHSS and linked with other stakeholders addressing HIV and AIDS.

5 Free and informed consent of the participants was obtained and the study protocol was approved by the Ethics Committee of the Office of Permanent Secretary, Ministry of Health and Social Services, Windhoek, Namibia on 12th March 2012 [Reference 17/3/3].

6 Home-based counselling and care for people with HIV and AIDS is provided at home by immediate family and friends, as well as by home-based care organisations. Providing care at home has many potential benefits for both HIV infected and affected people.

7 In 2008 there was an outbreak of cholera during flooding, in 2009 of meningitis and in 2010 of measles.

8 Defaulter tracing not only addresses HIV medication adherence, but also prevention of mother-to-child transmission (PMTCT) and tuberculosis (TB) treatment. The defaulting or abandonment of TB medication is very problematic, as patients remain infectious for longer, are more likely to relapse or succumb to tuberculosis. This could result in treatment failure as well as foster emergence of drug resistant tuberculosis (27).
